# Population genetic patterns across the native and invasive range of a widely distributed seagrass: Phylogeographic structure, invasive history and conservation implications

**DOI:** 10.1111/ddi.13803

**Published:** 2024-03-01

**Authors:** Xiaomei Zhang, Yu-Long Li, James E. Kaldy, Zhaxi Suonan, Teruhisa Komatsu, Shaochun Xu, Min Xu, Feng Wang, Peng Liu, Xujia Liu, Shidong Yue, Yu Zhang, Kun-Seop Lee, Jin-Xian Liu, Yi Zhou

**Affiliations:** 1 CAS Key Laboratory of Marine Ecology and Environmental Sciences, Institute of Oceanology, Chinese Academy of Sciences, Qingdao, China; 2 Laboratory for Marine Ecology and Environmental Science, Qingdao National Laboratory for Marine Science and Technology, Qingdao, China; 3 Center for Ocean Mega-Science, Chinese Academy of Sciences, Qingdao, China; 4 US EPA, Pacific Ecological Systems Division, Newport, Oregon, USA; 5 Department of Biological Sciences, Pusan National University, Pusan, Korea; 6 Japan Fisheries Resource Conservation Association, Tokyo, Japan; 7 University of Chinese Academy of Sciences, Beijing, China; 8 Guangxi Key Laboratory of Marine Environmental Science, Guangxi Academy of Marine Sciences, Guangxi Academy of Sciences, Nanning, China

**Keywords:** genetic differentiation, genetic diversity, invasion history, phylogeographic structure, seagrass, *Zostera japonica*

## Abstract

**Aim:**

The seagrass *Zostera japonica* is a dramatically declined endemic species in the Northwestern Pacific from the (sub)tropical to temperate areas, however, it is also an introduced species along the Pacific coast of North America from British Columbia to northern California. Understanding the population’s genetic patterns can inform the conservation and management of this species.

**Location:**

North Pacific.

**Methods:**

We used sequences of the nuclear rDNA internal transcribed spacer (ITS) and chloroplast *trn*K intron maturase (*matK*), and 24 microsatellite loci to survey 34 native and nonnative populations (>1000 individuals) of *Z. japonica* throughout the entire biogeographic range. We analysed the phylogeographic relationship, population genetic structure and genetic diversity of all populations and inferred possible origins and invasion pathways of the nonnative ones.

**Results:**

All markers revealed a surprising and significant deep divergence between northern and southern populations of *Z. japonica* in the native region separated by a well-established biogeographical boundary. A secondary contact zone was found along the coasts of South Korea and Japan. Nonnative populations were found to originate from the central Pacific coast of Japan with multiple introductions from at least two different source populations, and secondary spread was likely aided by waterfowl.

**Main Conclusions:**

The divergence of the two distinct clades was likely due to the combined effects of historical isolation, adaptation to distinct environments and a contemporary physical barrier created by the Yangtze River, and the warm northward Kuroshio Current led to secondary contact after glacial separation. Existing exchanges among the nonnative populations indicate the potential for persistence and further expansion. This study not only helps to understand the underlying evolutionary potential of a widespread seagrass species following global climate change but also provides valuable insights for conservation and restoration.

## 1 | INTRODUCTION

Genetic diversity is essential for populations to respond and adapt to environmental changes and plays an important role in reducing the extinction of populations associated with inbreeding (Frankham, 2005) and facilitating the establishment and colonization of populations outside their native range (Facon et al., 2006; Lavergne & Molofsky, 2007). Management of threatened and invasive species through the application of genetic principles is a top priority in conservation biology (Frankham, 2010). Elucidating the spatial and temporal distribution patterns of genetic diversity has proven to be a useful approach for identifying populations at risk (Yoder et al., 2018), defining conservation units (Barbosa et al., 2018) and retracing the sources and pathways of introduction (Estoup & Guillemaud, 2010). Taken together, a better understanding of evolution and genetic diversity can inform the management of threatened and invasive species.

Seagrasses are marine flowering plants that inhabit shallow coastal waters along all oceans and support one of the most productive ecosystems on earth (Fourqurean et al., 2012; Short et al., 2007; Unsworth, McKenzie, et al., 2019). Seagrass meadows all over the world have been suffering from dramatic declines due to a combination of natural and anthropogenic impacts (Orth et al., 2006; Waycott, 2009). In particular, the North-Western Pacific (NWP) region, including China, Korea, and Japan, has the highest number of threatened or near-threatened seagrass species in the world (Short et al., 2011). Consequently, seagrass conservation and restoration has become a global concern (Pazzaglia et al., 2021; Unsworth, Nordlund, et al., 2019; van Katwijk et al., 2009). In this context, seagrass population genetic studies have increased dramatically since the early 20th century (Procaccini et al., 2007). Furthermore, these studies highlight the role of genetic diversity in enhancing seagrass resilience (Hughes & Stachowicz, 2004) and restoration success (Unsworth et al., 2012). However, population genetic information for NWP seagrass species is still limited in comparison with the well-documented congener species or populations along the coasts of Europe, Australia and the Atlantic Coast of North America (Procaccini et al., 2007).

*Zostera japonica* is unique as one of the only two seagrass species (the other one is *Halophila stipulacea*) considered both endemic and ‘invasive’ (Shafer et al., 2014). *Zostera japonica* is endemic to the NWP, and distributed across a broad latitudinal range from Vietnam at ~10° N to the Russian Federation at ~55° N (Green & Short, 2003; Shin & Choi, 1998). The native populations of *Z. japonica* have been suffering from severe degradation due to frequent anthropogenic disturbances and extreme climate events, such as dredging, reclamation, clam harvesting (Huang et al., 2006; Lee, 1997; Park et al., 2011), invasion of Salt marsh (Yue, Zhou, et al., 2021) and typhoon (Yue, Zhang, et al., 2021).

These natural and anthropogenic stressors have resulted in widespread degradation and loss of *Z. japonica* meadows to the point that it is now considered ‘threatened or endangered’ in its native range (Hodoki et al., 2013; Lee et al., 2004). In contrast, *Z. japonica* was introduced to the Pacific Coast of North America (PNA) in the early 20th century and successfully colonized estuarine tidal flats. It was first reported from Willapa Bay in 1957 (Hitchcock et al., 1969) and subsequently found in various bays and estuaries from southern British Columbia to northern California within the subsequent 50 years (Shafer et al., 2014). Successful colonization and expansion of *Z. japonica* have caused concerns about its ecological effects on ecosystem services and economic impacts on oyster aquaculture (Shafer et al., 2014). The dual nature of endemic and invasive populations leads to a conservation paradox (Marchetti & Engstrom, 2016) where a growing body of effort is devoted to the conservation and restoration of *Z. japonica* in its native areas while competing policies in the nonnative range vary among protection, neutrality and eradication (Shafer et al., 2014).

The NWP is characterized by unique tectonic and intricate hydrologic features (Wang, 1999) and abundant marine biodiversity (Keith et al., 2014; Liu, 2013; Sun et al., 2016). Modern phylogeographic work in NWP has mostly focused on animals (molluscs, fishes and crustaceans) and a few algae (Ni et al., 2014). Intraspecific evolutionary history of the marine species inhabiting the NWP has been deeply influenced by the isolation effect of the marginal seas, notably the Sea of Japan/East Sea, Yellow-Bohai Sea, East China Sea and South China Sea, during ice ages (Ni et al., 2014), and by the barrier effect of the Yangtze River outflow (Ni et al., 2017), as well as the population connectivity driven by Kuroshio current or coastal currents (Hu et al., 2011, 2013; Li et al., 2017). In addition, increasing evidence suggests that temperature gradients have also contributed substantially to the accelerating rate of intraspecific differentiation and sister-/sub-/cryptic speciation in NWP (Lee et al., 2021; Ni et al., 2015; Takeuchi et al., 2020; Zhao et al., 2017). As the genetic population structure of *Z. japonica* is only available at local or regional scales (Araki & Kunii, 2006; Hodoki et al., 2013; Jiang et al., 2018, 2020; Lee et al., 2004; Zhang et al., 2016), a comprehensive understanding of the full picture of the population structure of this widespread species is still lacking. Additionally, it is unclear whether and how the complex tectonic, hydrologic and other environmental features in NWP have influenced the contemporary population structure of *Z. japonica* in its native range.

Hundreds of marine species have been introduced to PNA in recent centuries (Wonham & Carlton, 2005). Transportation of the Pacific oyster (*Crassotrea gigas*) from Japan has been proposed to be the primary vector facilitating the introduction of nonnative invertebrates (Quayle, 1988) and algae (Scagel, 1956) to PNA. To date, only two studies have attempted to retrace the invasion history of estuarine invaders associated with Japanese oyster *C. gigas* based on molecular markers (Krueger-Hadfield et al., 2017; Miura et al., 2006). The *Z. japonica* introduction also aligns with Japanese oyster transportation in the early 20th century (Harrison & Bigley, 1982). Evidence supporting the introduction of *Z. japonica* from the coasts of Japan has been largely derived from ecological and economic data, but lacks genetic evidence. Moreover, important information for establishing effective management strategies, including source populations, population connectivity and genetic diversity, is still unresolved, preventing the full understanding of invasion history and further invasive potential of *Z. japonica*.

To investigate the phylogeographic and invasion history of *Z. japonica*, we conducted the first large-scale population genetic study on *Z. japonica* by sampling both its native and nonnative ranges and using a combination of chloroplast and nuclear DNA sequences and microsatellites. For a species that is both endangered and invasive, comparison of native populations with introduced ones may provide an informative contrast, which would be instructive for management of both the native and nonnative populations.

## 2 | METHODS

### 2.1 | Sampling and DNA extraction

**FIGURE 1:**
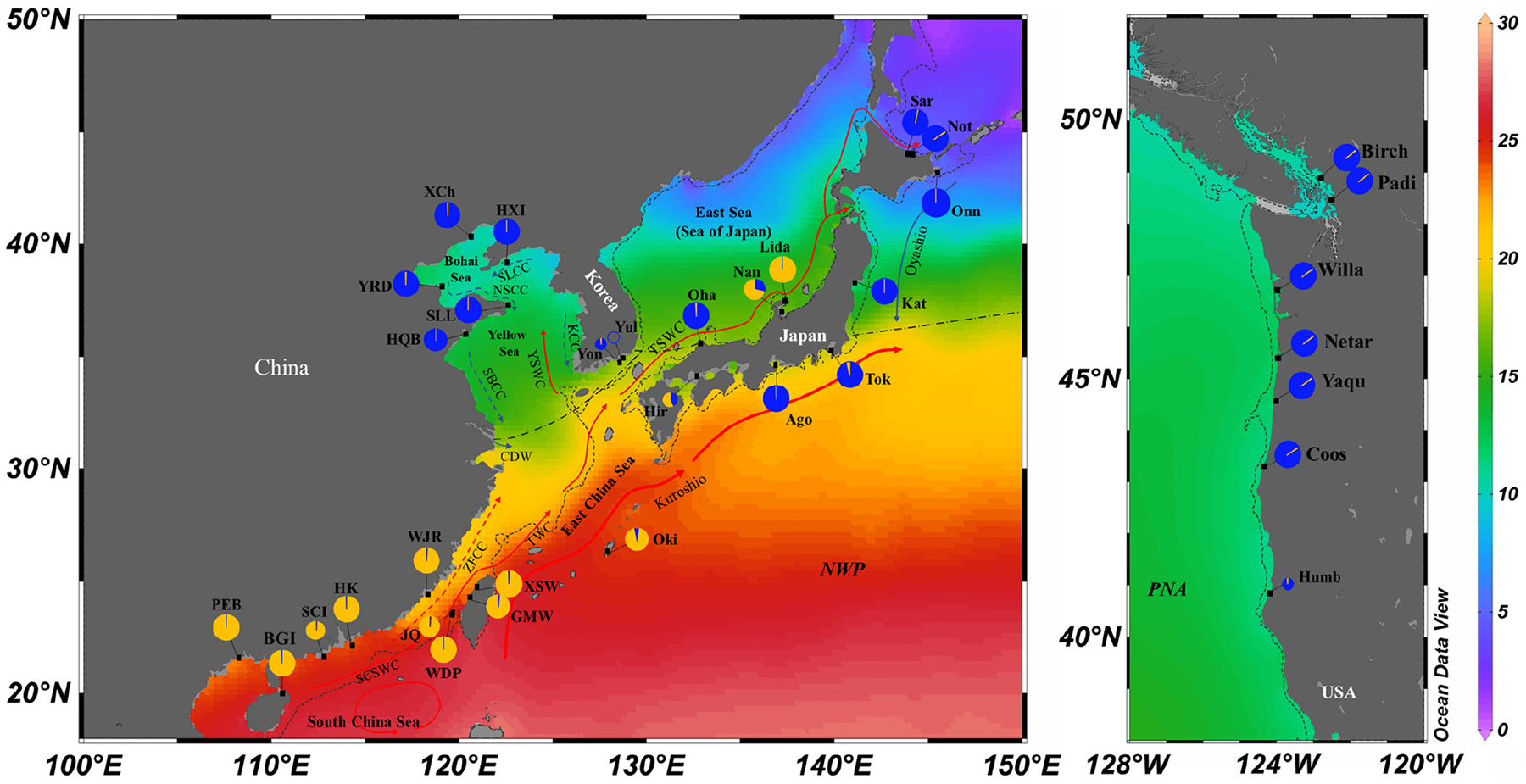
Sample locations and the distribution of the two genetic clades of *Zostera japonica* inferred from STRUCTURE analysis (best *K* = 2) based on 24 microsatellite loci. The left and right panels represent native and invasive ranges respectively. The southern and northern clades are represented by orange and blue coloured circles respectively. The map also presents the annual mean sea surface temperature distribution across the study area, as derived from the World Ocean Atlas 2018 dataset (Locarnini et al., 2019) using Ocean Data View software (Schlitzer, 2015). Currents in summer are depicted with arrows and include the South China Sea Warm Current (SCSWC), Taiwan Warm Current (TWC), MinZhe Coastal Current (ZFCC), Changjiang Diluted Water (CDW), Subei Coastal Current (SBCC), Lubei Coastal Current (LBCC), Liaonan Coastal Current (LNCC), Yellow Sea Warm Current (YSWC) and Tsushima Warm Current (TSWC). The shelf outcrop of the last glacial maximum is represented by a dotted line. The dash/dotted line separates two biogeographic regions: the North Pacific Temperate Biotic Region (above the line) and the Indo-West Pacific Warm Water Biotic Region (below the line).

Between 2012 and 2020, we collected 15–32 individual samples of *Z. japonica* per population from each of 27 native populations (806 individuals) across the Asian coastlines (China, South Korea and Japan), and 7 nonnative populations (209 individuals) along the Pacific coast of North America, USA (for sampling information, see the Table S1.1 and Figure 1). Shoots connected by the same rhizome were considered one sample and were collected by walking or wading during the low tide. Distance between sampling points ranged between 30 cm and >20 m depending on the meadow area or coverage (continuous vs. patchy). Fresh leaves, sheaths and rhizomes were preserved in silica gel or in plastic bags in a cooler on ice in the field and then subsequently stored at −80°C until processed for DNA extraction. Genomic DNA was extracted using an HP plant DNA kit (OMEGA Biotek) following the manufacturer’s protocols.

### 2.2 | Microsatellite genotyping and genet determination

All DNA samples were genotyped using 24 microsatellite loci (ZJ008, 011, 018, 025, 026, 028 and 042; Zj1293493, 501127, 2574387, 177154, 1954829, 887337, 2125746, 1833855, 1141525, 1332296, 1967651, 3450072, 974910, 109976, 2349254, 599480 and 1098720) as outlined in the literature (Zhang et al., 2015; Zhang, Zhou, et al., 2019). Allele scoring was performed using GeneMarker 2.2.0 (SoftGenetics) and double checked manually. Only individuals with missing microsatellite loci ≤2 were included, resulting in retention of 759 native and 207 nonnative samples respectively. Genclone 2.0 (Arnaud-Haond & Belkhir, 2007) was used to identify the multilocus genotypes (MLG) shared by more than one ramets. The statistic *P*_sex_ was calculated for each pair of samples to examine whether the ramets sharing identical genotypes arise from clonal replicates or independent sexual reproduction events. If a probability *P*_sex_ < .01, it indicates that the ramets come from the same clone (genet) and only one sample per MLG group (genet) was used in the subsequent analyses. In addition, a large number of (26 of 32) samples from population Yul (Korea) showed more than two alleles on most (17 of 24) of the microsatellite loci, which may have resulted from polyploidy (Edgeloe et al., 2022) or somatic mutation (Reusch & Boström, 2011). Hence, this population was excluded from the subsequent microsatellite analysis.

### 2.3 | Direct sequencing and cloning of PCR products

Based on the genet dataset yielded by microsatellites, specimens of *Z. japonica* were randomly selected from each population to amplify chloroplast-encoded *matK* intron, and almost every population, except for three (Yon, Hir and Sar) with insufficient specimens, then to amplify nuclear rDNA ITS (Table S1.1), following established procedures (Zhang et al., 2016). When direct sequencing of the ITS fragment failed due to heterogeneity among tandem copies (Harris & Crandall, 2000), the amplicons were ligated to the plasmid vector pESI-T vector via the Hieff Clone^®^ Zero TOPO TA-cloning kit (Yeasen Biotechnology) and sequenced using M13 primers. Five colonies were chosen for each PCR reaction (individual). A total of 303 and 279 samples were successfully sequenced for *matK* and ITS respectively. The *matK* and ITS sequences obtained in this study and the already published sequences for six Chinese populations (YRD, SLL, HQB, HK, BGI and PEB) were pooled together for subsequent analysis (Zhang et al., 2016).

### 2.4 | Sequence genetic variation and network

Sequences were edited using DNASTAR software (DNASTAR, Inc.) and then aligned with BioEdit 7.053 (Hall, 1999). To investigate the genealogical relationships among *matK* haplotypes and ITS genotypes, the minimum spanning network approach (Bandelt et al., 1999) was used as implemented in PopART (Leigh & Bryant, 2015). A maximum-likelihood (ML) tree was constructed based on ITS haplotype sequences using IQ-TREE v2.2.0 (Nguyen et al., 2015) with 1000 ultrafast bootstraps.

### 2.5 | Microsatellite population genetic structure

#### 2.5.1 | Bayesian clustering

We employed a Bayesian clustering approach to investigate spatial genetic structures using STRUCTURE 2.3.4 (Pritchard et al., 2000), incorporating an admixture model with correlated allele frequencies (Falush et al., 2003). Five datasets were analysed successively according to their hierarchical levels: (1) global populations were analysed with *K* values varying from 1 to 20; (2) as two deeply divergent clades (Clades N and S) identified by sequencing, the 12 native populations belonging to Clade N were independently analysed with *K* values varying from 1 to 13; (3) the 13 native populations belonging to Clade S were independently analysed with *K* values varying from 1 to 14; and (4) the seven nonnative populations were independently analysed with *K* values varying from 1 to 8; and (5) the 7 nonnative populations and the 6 most possible source populations were pooled together to be analysed with *K* values varying from 1 to 14. For each analysis, 10 independent runs were carried out for each *K* value with no prior knowledge of sampling locations. Each run involved Markov Chain Monte Carlo (MCMC) procedure with 10^6^ iterations, following a burn-in period of 2 × 10^5^ iterations. The Structure Selector (Li & Liu, 2018) was used to determine the optimal *K* based on the Delta *K* method (Evanno et al., 2005) and visualized the output of the STRUCTURE analysis using CLUMPAK (Kopelman et al., 2015).

#### 2.5.2 | Multivariate analyses

Discriminant analysis of principal components (DAPC) was applied to examine the genetic relationships among populations at different geographic scales by using the *R* package *adegen*et 2.0.1 and the best PC was chosen following the alpha-score indication (Jombart, 2008). A DAPC analysis is a multivariate, model-free approach designed to cluster based on prior population information (Jombart et al., 2010). The same five datasets mentioned above were analysed, respectively: (1) global populations; (2) the native populations corresponding to Clade N; (3) the native populations corresponding to Clade S; (4) only nonnative populations; and (5) nonnative populations and their possible source populations. For the DAPC plot, inertia ellipses were generated encompassing the conventional ~67% of the cloud of points for each sampling location.

#### 2.5.3 | Neighbour-joining (NJ) tree

To obtain a more explicit phylogenetic perspective of the genetic relationship between populations, we utilized POPTREE2 (Takezaki et al., 2010) to construct a neighbour-joining (NJ) tree (Saitou & Nei, 1987) based on DA distance (Nei et al., 1983). One thousand bootstrap replicates were used to estimate branch support.

#### 2.5.4 | Genetic differentiation

Population genetic differentiation was estimated by calculating the pairwise *F*_ST_ values in ARLEQUIN 3.5 (Excoffier & Lischer, 2010), and significance of each pairwise *F*_ST_ value was assessed by 10,000 permutations.

#### 2.5.5 | Migration rate and isolation by distance

We used BayesAss 3.0.4 to estimate *Z. japonica* recent migration rate (m) over the last several generations among populations using a Bayesian method with Markov Chain Monte Carlo (MCMC) simulations (Wilson & Rannala, 2003). We performed 10 independent simulations (2 × 10^7^ iterations, 10^7^ burn-in and sampling frequency of 2000) for each of the 5 previously described datasets, and each run was initialized with a different seed. Mixing parameter values for migration rate, allele frequency and inbreeding coefficients were adjusted to achieve acceptance rates of 20%–40%. To determine the best-fit run, we calculated a Bayesian deviance measure for each run using the R script of Meirmans (2014) and selected the run with the lowest deviance to report the results (Faubet et al., 2007). To test for isolation by distance (IBD), a Mantel test of linearized genetic distances (*F*_ST_/(1 − *F*_ST_)) versus geographic distances was separately performed for Clade N, Clade S and USA group in IBDWS 2.10 (Jensen et al., 2005) with 10,000 randomizations. Geographic distances between sampling sites were estimated as minimum coastline distances by using Google Maps.

### 2.6 | Microsatellite genetic diversity

Microsatellite stuttering and large allele dropout rates were detected using Micro-checker 2.2.3 (Van Oosterhout et al., 2004), and the presence of null alleles was checked using FreeNA (Chapuis & Estoup, 2007). Genotypic linkage disequilibrium (LD) and deviations from Hardy–Weinberg equilibrium (HWE) were evaluated globally and across all populations individually using Genepop 4.7 (Rousset, 2008) with significance levels adjusted for multiple comparisons following a standard Bonferroni correction. Genotypic/clonal richness was estimated as *R* = (G−1)/(*N*−1), where *G* is the number of genets and *N* is the number of ramets sampled for a population. Allelic richness (*A*_R_) was determined with FSTAT 2.9.3 (Goudet, 2001). The number of alleles per locus (*N*_a_), the observed and expected heterozygosity (*H*_*O*_ and *H*_*E*,_ respectively), private alleles (*P*_A_) and inbreeding coefficient (*F*_IS_) were calculated using GENALEX 6.5 (Peakall & Smouse, 2012). Nonparametric tests on independent samples were performed in SPSS23.0 to compare the estimates of genotypic (*R*) and genetic diversity (*H*_*O*_, *H*_*E*_, *N*_a_, AR and *P*_A_) between native populations in Clade N (NWP-N) and S (NWP-S), and nonnative populations in the USA. Populations with signs of an admixture of Clades N and S (Nan and Hir) and with small sample sizes (Yon and Humb; *G* = 6) were excluded from this comparison. Effective population sizes (*N*_e_) were evaluated for each population using the linkage disequilibrium model with random mating (Waples & Do, 2008) implemented in NeEstimator version 2.1 (Do et al., 2014). We set the critical value *P*_crit_ = .05 to filter out rare alleles and generated 95% confidence intervals using the Jackknife across samples method (Jones et al., 2016).

## 3 | RESULTS

### 3.1 | Sequences variation and network

#### 3.1.1 | matK

**FIGURE 2:**
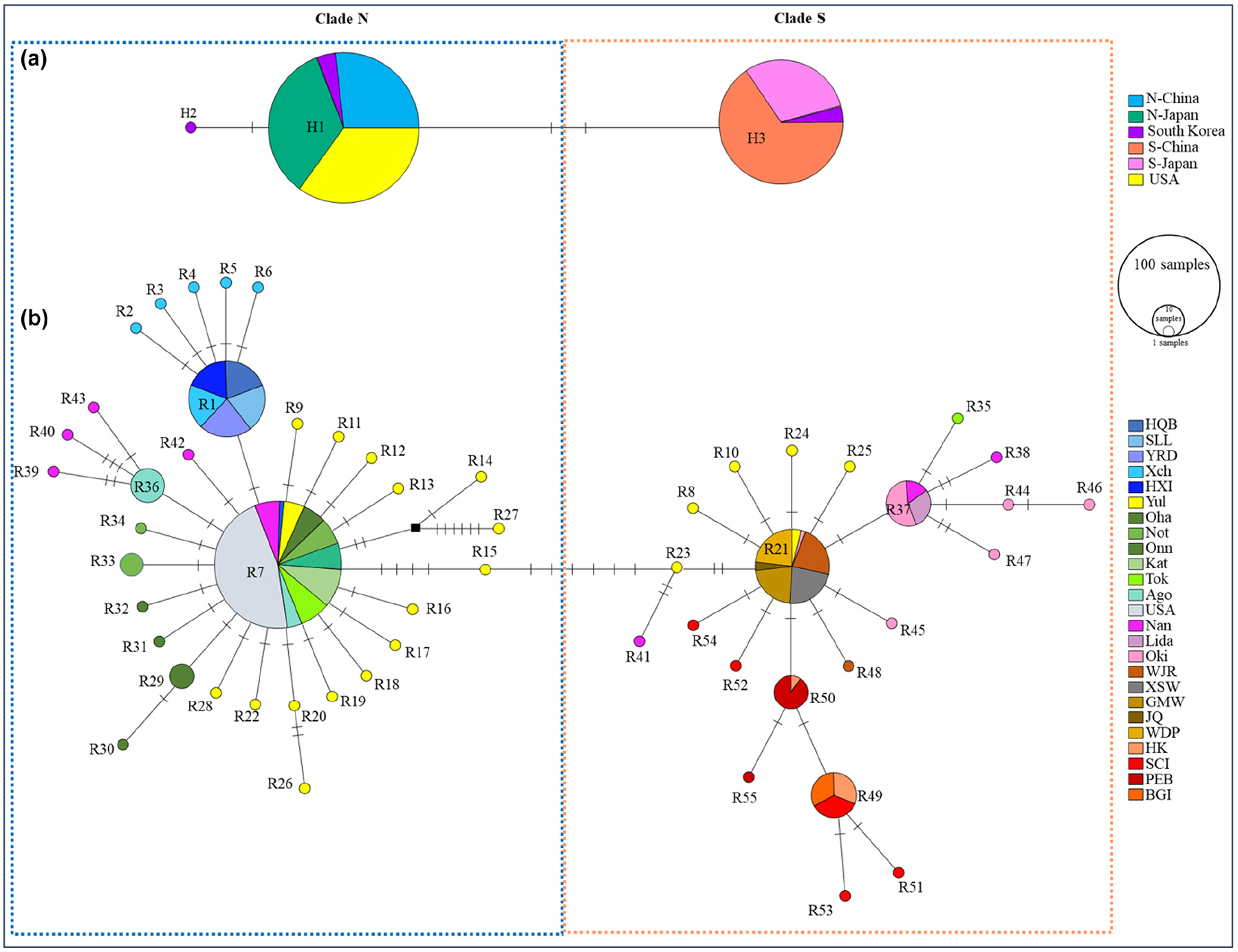
The minimum-spanning network constructed in PopART for the 3 cp *matK* haplotypes (a) and 55 nrITS ribotypes (b) of *Zostera japonica*. Each circle represents one unique haplotype/ribotype, and colours indicate geographic regions or sample locations. The sizes of the circles are proportional to the number of individuals. Mutational steps between haplotypes/ribotypes are indicated by hatch marks.

Only three variable sites were identified in *matK* across an aligned length of 639 bp, including one singleton and two parsimony informative sites. This defined three haplotypes (H1, H2 and H3; Figure 2a), based on which all populations were grouped into two clades separated from each other by two mutational steps (hereafter referred to as Clade N and Clade S, Figure 2a). Clades N and S were dominated by haplotypes H1 and H3, respectively, and geographically structured in its native range (Figure S2.1A). Clade N dominated the northern temperate zone, including the northern part of China (Yellow-Bohai Sea), northern to central Pacific Japan and some areas in Sea of Japan/East Sea and South Korea, while Clade S dominated the southern subtropical zone, including the South China Sea, East China Sea (restricted to the Taiwan Strait), Okinawa, Inland Sea and some areas in Sea of Japan/East Sea and South Korea (Figure S2.1A). We then divided the Chinese populations into two groups based on clade assignments, namely Clade N-CN and Clade S-CN (Figure 2a). Similarly, the Japanese populations were divided into Clade N-JP and Clade S-JP (Figure 2a). Interestingly, the Korean population Yul showed co-occurrence of both clades. Moreover, only haplotype H1 was detected in the USA, suggesting that the American populations were derived from Clade N.

#### 3.1.2 | ITS

A total of 402 ITS sequences with a length of 635 bp, consisting of partial ITS1 (216 bp), complete 5.8 S (159 bp) and partial ITS2 (260 bp), were used for analysis. Among these, 72 ITS sequences were obtained from our published data for six Chinese populations (YRD, SLL, HQB, HK, BGI and PEB; Zhang et al., 2016; Table S1.1). The other 330 ITS sequences were newly sequenced through direct sequencing or TA cloning of 279 specimens (Table S1.1). A total of 23 of the 279 specimens could not be directly sequenced and thus were subjected to TA cloning. The number of ribotypes (alleles) per specimen in the 23 cloned specimens was in most cases larger than two (Table S1.2), indicating intragenomic variations (i.e. variable repetitive sequences). Moreover, the sequences obtained from the same individual were not all monophyletic. We observed co-existence of ITS copies belonging to both Clades N and S within six individuals, with three samples from South Korea (population Yul) and the other three from Japan (one from population Tok and two from population Nan; Table S1.2). We found 71 polymorphic sites, including six short indels, which defined 55 ribotypes, among which 46 were singletons present in only one individual. The minimum-spanning network of ITS ribotypes resolved congruent genetic groups (N, S) with *matK* and revealed further substructure (Figure 2b). In general, the two ITS clades were allopatrically distributed within the native range of *Z. japonica*, while overlapped in one Korean population (Yul) and two Japanese populations (Nan and Tok; Figure S2.1B). Clade N consisted of 33 ribotypes, forming a star-like network with a dominant ribotype R7 in the centre. Ribotype R1, connected to R7 by one mutational step, dominated the Chinese populations in the Yellow-Bohai Sea. In contrast, the 22 ribotypes of Clade S formed a more variable network with four major ribotypes (R21, R37, R49 and R50), which largely corresponded to different geographic regions, including the Taiwan Strait (R21), Oki and part of the Sea of Japan/East Sea (R37), and the South China Sea (R49 and R50; Figure 2b). Moreover, the ribotypes R49 and R50 were separately distributed on the west and east sides of Qiongzhou Strait. Similar to the results based on *matK*, only one single ribotype (R7) was identified in American populations, suggesting that the invasive sources were most likely from Clade N-JP. The ML phylogenetic tree of ITS produced a consistent structure with the minimum-spanning network (Figure S2.2).

### 3.2 | Microsatellite population genetic structure

#### 3.2.1 | Global structure

**FIGURE 3:**
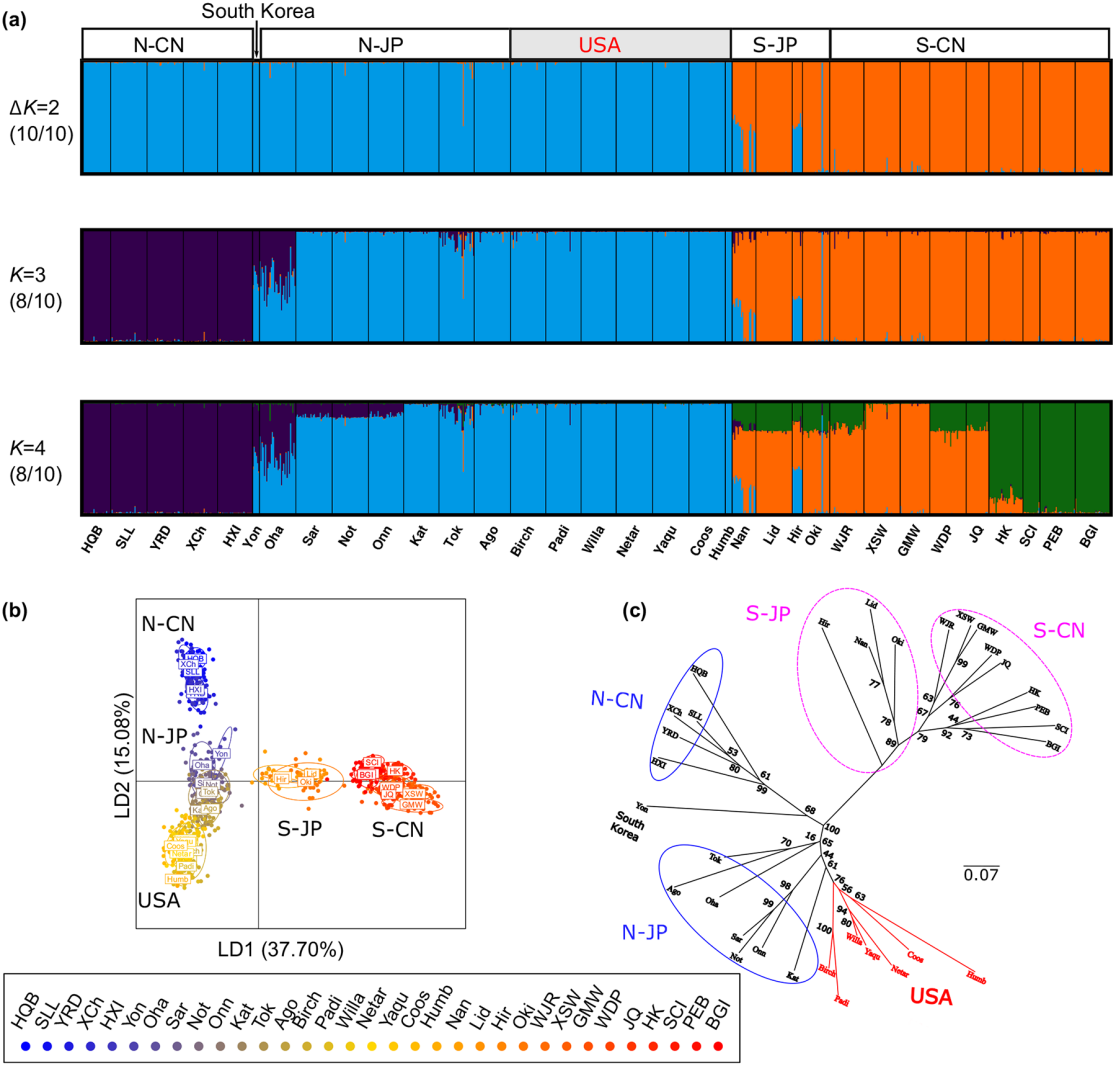
Global genetic population structure of *Zostera japonica* based on 24 microsatellite loci. (a) STRUCTURE bar plots for *K* = 2, 3 and 4 respectively. (b) Scatterplots of DAPC based on the first two main components. (c) Neighbour-joining (NJ) tree.

When *K* = 2, STRUCTURE analysis of all the populations supported the division of two clades revealed by *matK* and ITS (Clade N and Clade S, Figure 3a). When *K* = 3, Clade N-CN and N-JP were further separated from each other, with admixtures observed in one Korean population (Yon) and one Japanese population (Oha). Multivariate analyses using DAPC demonstrated a similar global grouping to the STRUCTURE analysis (Figure 3b). Clade N and Clade S were separated by the first discriminant function, and the second discriminant function further separated N-CN and N-JP. The populations from the USA consistently clustered with N-JP in both the STRUCTURE and DAPC analyses (Figure 3a,b). The geographical distribution pattern of the two clades defined by microsatellites (Figure 1) was consistent with those by *matK* and ITS (Figure S2.1).

#### 3.2.2 | Substructure in the native range

There was clear substructure within both Clade N and Clade S in the native range (Figure S2.3). In Clade N, three genetically distinct subgroups were defined by STRUCTURE analysis at the optimal *K* = 3 (Figure S2.3A), namely N-CN and two subgroups of N-JP (I: Sar + Not+Onn + Kat; and II: Oha + Ago+Tok). Admixture of the two subgroups of N-JP was observed in one Korean population (Yon, Figure S2.3A). The first two discriminant functions of DAPC analysis also identified three subgroups (Figure S2.3A), including N-CN and two subgroups of N-JP (I: Sar + Not+Onn; and II: Oha + Ago+Tok + Kat). The division of the two subgroups of N-JP was not completely consistent between STRUCTURE and DAPC analyses, indicating the genetic complexity of Japanese populations. For Clade S, the optimal *K* value was extremely high compared to the total number of populations (11 vs. 13), and was not informative (Figure S2.3B). In contrast, the STRUCTURE mode at *K* = 3 revealed a grouping that was consistent with DAPC (Figure S2.3B).

#### 3.2.3 | Structure in the nonnative range

**FIGURE 4:**
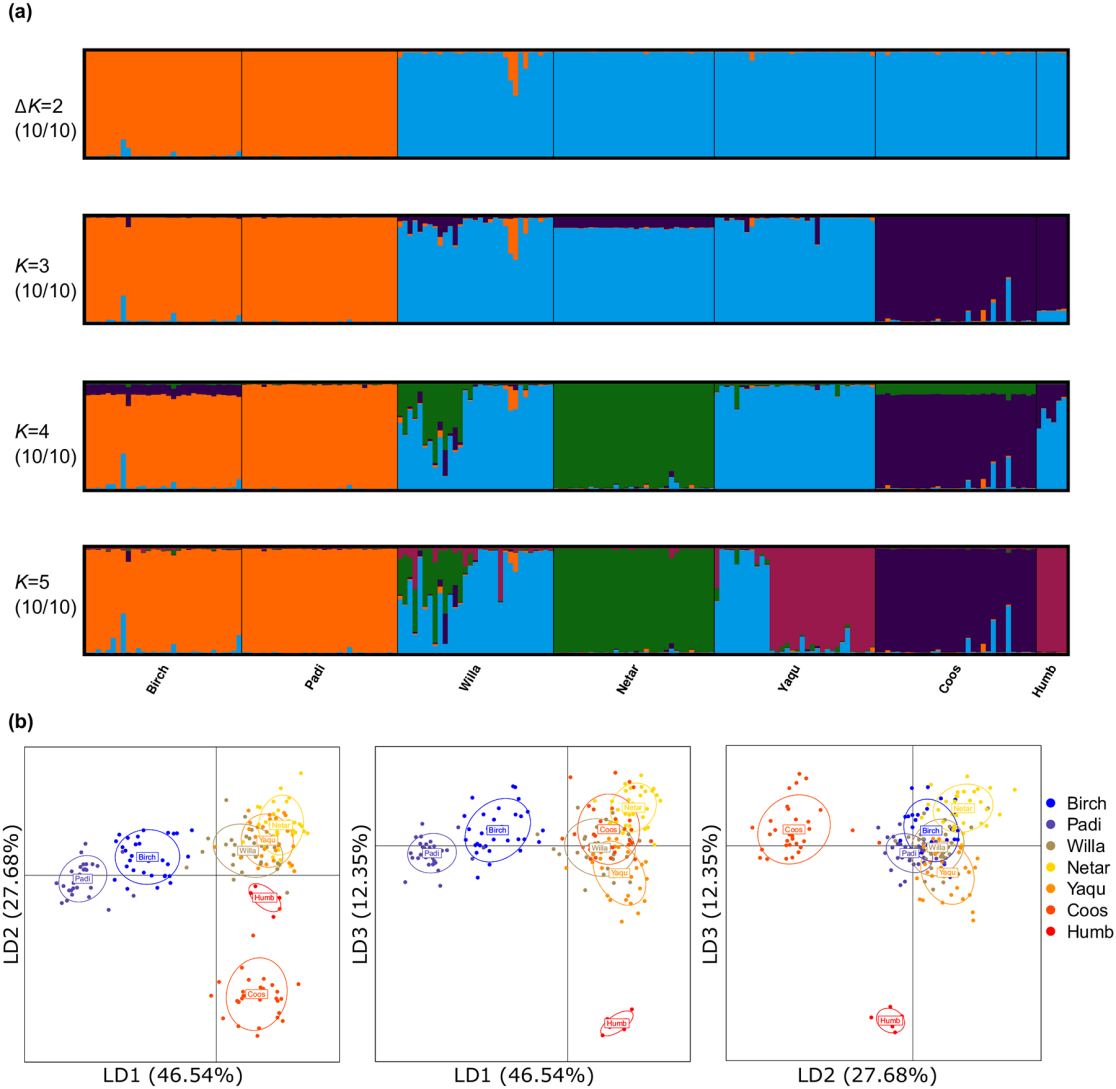
Genetic population structure of *Zostera japonica* populations across its nonnative range based on 24 microsatellite loci. (a) STRUCTURE bar plots for *K* = 2 to 5. (b) Scatterplot of DAPC based on the first three discriminant functions.

An independent STRUCTURE analysis for all the American populations revealed two genetic groups (hereafter n-USA and s-USA) at the optimal *K* = 2 (Figure 4a), representing the highest hierarchical structure. Group n-USA included the two northernmost populations (Birch and Padi), while s-USA was composed of the other five populations. Further genetic substructure was successively revealed with increasing *K* values (Figure 4a). Group n-USA remained the same for *K* = 2 to 5. Group s-USA diverged into two subgroups when *K* = 3. When *K* ≥ 4, substructure was observed within populations Willa and Yaqu. The reason could be that the samples of those two populations were collected from 2–3 spatially distinct sites within the estuaries (Table S1.1). When running DAPC analysis on all American populations, the same two groups identified by STRUCTURE analysis were separated by the first discriminant function (Figure 4b). Furthermore, DAPC analysis identified three distinct genetic groups (Figure 4b), which largely coincided with the results of STRUCTURE analysis (*K* = 3) except for the population Coos.

#### 3.2.4 | Structure of the possible source and nonnative populations

**FIGURE 5:**
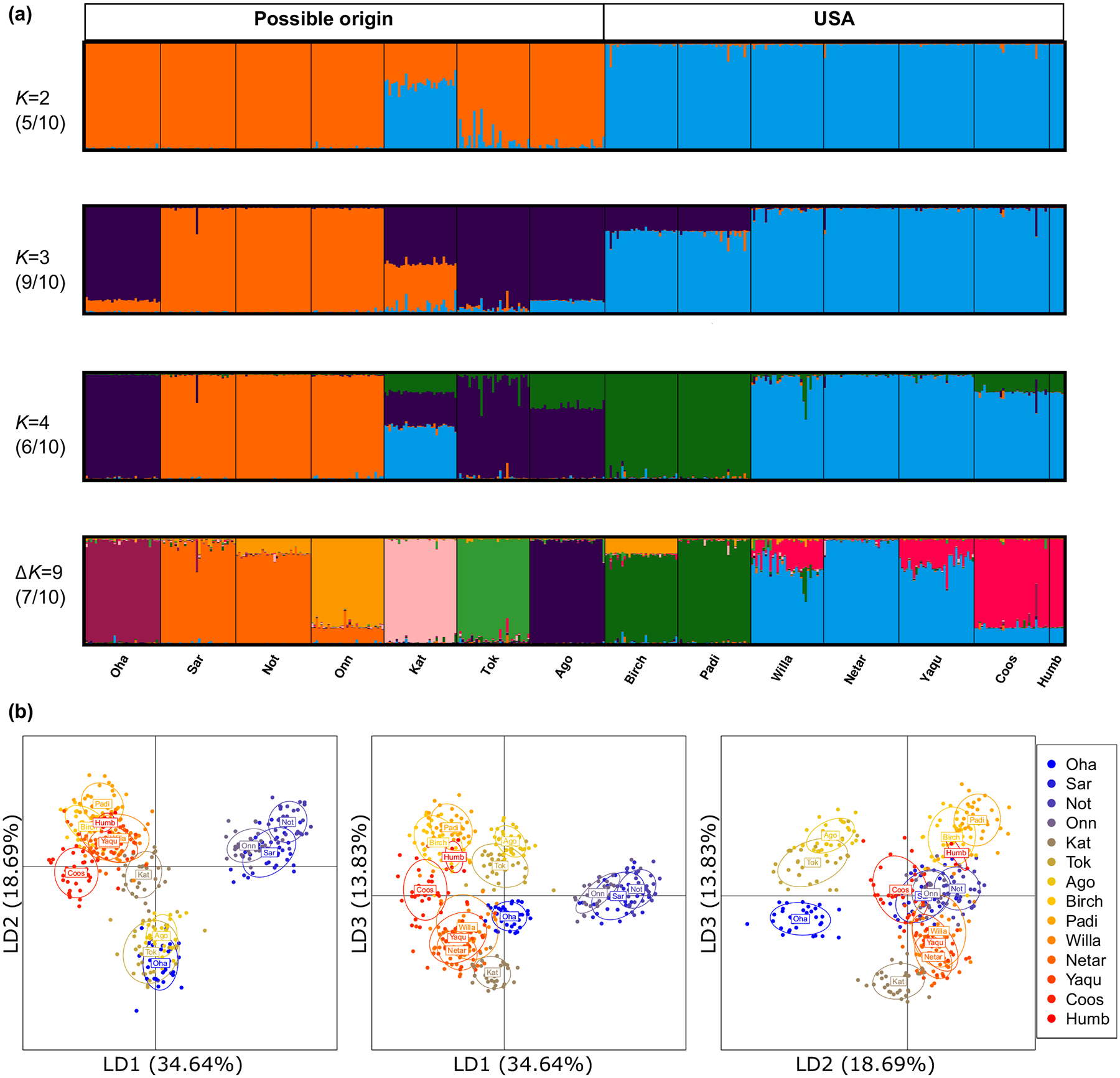
Genetic population structure across the possible origins and nonnative populations. (a) STRUCTURE bar plots for *K* = 2, 3, 4 and 9. (b) Scatterplots of DAPC based on the first three discriminant functions.

Based on all the above results, we inferred that nonnative populations were most likely derived from Clade N-JP. Hence, we conducted an independent analysis for N-JP and American populations (Figure 5). When *K* = 2, STRUCTURE analysis revealed that the American genetic component dominated the population Kat. Population Tok also shared genetic component with USA, but only with a very small proportion (Figure 5a). The results became complicated with the increasing *K* values, for instance, at *K* = 3, 4 and 9 (Figure 5a). As for DAPC analysis, the first two discriminant functions showed that the American populations clustered most closely with population Kat, consistent with the STRUCTURE results for *K* = 2 (Figure 5b). DAPC analysis also showed that Hokkaido populations, Sar, Not and Onn, always clustered together, forming an independent group that was relatively distant from the American populations, which indicated that the Hokkaido populations were not likely to be the source populations (Figure 5b).

#### 3.2.5 | Neighbour-joining (NJ) tree

All the native populations clustered into four groups with bootstrap value ≥79, including N-CN, N-JP, S-JP and S-CN, which was well corresponded to the results of DAPC analysis at the global range (Figure 3c). The American populations were clustered into the same three clusters as identified in the STRUCTURE analysis with *K* = 3 (Figure 4a). Moreover, the American populations were closest to population Kat belonging to Clade N-JP, which was consistent with the results of STRUCTURE and DAPC analysis (Figure 5).

#### 3.2.6 | Pairwise *F*_ST_

Pairwise *F*_ST_ displayed significant differentiation among all populations (*p* < 10^−5^; Table S1.3). Within the native areas, pairwise *F*_ST_ values averaged 0.365, ranging from 0.108 to 0.673. The *F*_ST_ between populations in Clade S averaged 0.415, ranging from 0.236 to 0.648, which was much higher than in Clade N with a mean of 0.261 and a range of 0.108 to 0.384. The *F*_ST_ between populations of the two clades averaged 0.445, with a range of 0.280 to 0.673. Within the nonnative areas, pairwise *F*_ST_ values were also significant and relatively high with an average of 0.250, ranging from 0.039 (Willa vs. Yaqu) to 0.507 (Padi vs. Humb). Between the native and nonnative populations, the pairwise *F*_ST_ varied greatly from 0.153 (Kat vs. Willa) to 0.806 (GMW vs. Humb). The *F*_ST_ between native Clade N and nonnative populations averaged 0.321, with the lowest between Kat and Willa (0.153) and second lowest between Tok and Willa (0.189).

#### 3.2.7 | Migration rate and IBD

Generally, the contemporary gene flow (*m*) between most populations across *Z. japonica’s* range was negligible (<0.01). However, significant asymmetrical migration rates (>0.1) were observed in four native population pairs (Lid-Nan: 0.1346, XSW-GMW: 0.1526, WDP-JQ: 0.1329; and BGI-SCI: 0.1107) and one nonnative population pair (Willa-Yaqu: 0.1712; Table S1.4). Mantel test showed a significant pattern of IBD within Clade N (*p* < .05), but not in Clade S or USA (*p* > .05; Figure S2).

### 3.3 | Microsatellite genetic diversity

No loci exhibited significant linkage disequilibrium or deviations from Hardy–Weinberg equilibrium across all populations. Linkage disequilibrium was observed in 155 of 15,246 locus–locus pairs (adjusted *p* < .0001), and most occurred in Nan (123 pairs) and HQB (24 pairs), likely because of the relatively high clonal diversity and thus possibly high proportions of parent–offsprings or siblings existing in the samples from the two meadows. Deviations from Hardy–Weinberg equilibrium were observed in 23 populations by 1–5 loci after Bonferroni correction (Table S1.5). No scoring errors or large allele dropouts were identified, and null alleles were not systematically observed for a given sample across all the markers or for a given marker across all samples.

Genotypic diversity (*R*) varied from 0.31 (Humb) to 1.00, with almost 80% of populations exhibiting high clonal diversity (*R* > 0.90) and only a few with lower diversity (*R* < 0.60; Table S1.6). No significant differences existed among groups NWP-N, NWP-S and USA (*p* > .05; Figure S2.5A). However, all the genetic diversity indices (*H*_E_, *H*_O_, *N*_A_ and *A*_R_) were significantly higher in the NWP-N group as compared to those of NWP-S as well as USA (*p* < .05; Figure S2.5B–E). Among these indices, *H*_O_ of the three groups were 0.61, 0.33 and 0.49, respectively, and *A*_R_ were 3.90, 2.46 and 3.20 respectively. The number of private alleles in the global range (*P*_A-Globe_) varied from 1 to 12 within NWP-N (sum = 73), from 0 to 16 within NWP-S (sum = 47) and from 0 to 4 within USA groups (sum = 12; Table S1.6). The *P*_A-Globe_ of NWP-N was not significantly different from NWP-S (*p* > .05), but significantly higher than that of USA (*p* < .05; Figure S2.5F). The highest number of private alleles in America (*P*_A-USA_) was observed in Padi (22), followed by the Coos (15) and Birch (14), while no private alleles were detected in Humb (Table S1.6).

Effective population size (*N*_e_) varied greatly among populations (Table S1.6). The averaged *N*_e_ for the native populations was 149.38 with a range from 1.2 to infinity, while the average of *N*_e_ for the nonnative populations was 45.60, ranging from 9.50 to infinity. Within its native range, most populations (9 of 13) in Clade N had a relatively high *N*_e_ (>100), while most populations (9 of 12) in Clade S had a much smaller *N*_e_ (<50). In particular, extremely low *N*_e_ (<10) were found in eight native populations and one nonnative population (Humb, *N*_e_ = 9.5).

## 4 | DISCUSSION

### 4.1 | Phylogeographic patterns in the native range

#### 4.1.1 | Deep divergence between Clades N and S

The phylogeographic structure of *Z. japonica* in NWP is characterized by two genetically and geographically divergent clades (Clades N and S), which were consistently supported by three types of markers. The distribution areas of the two clades were well separated corresponding to the temperate versus tropical-to-subtropical zones, with a narrow secondary contact zone along the coasts of South Korea and central to southern Japan. In comparison, five times more variable sites in the ITS2 were found in *Z. japonica* than in *Z. marina* (Olsen et al., 2004), which is widely distributed across the temperate coastlines along both the Pacific and the Atlantic Oceans in the north hemisphere. Recently, Yu et al. (2023) detected 151 SNPs throughout the whole chloroplast genome (143,968 bp) of *Z. marina*. Assuming the same substitution rate for both *Z. japonica* and *Z. marina*, it is expected to detect 0.67 SNP for the *matK* fragment (639 bp) in our study, which is much smaller than our observed value (i.e. three sites). Thus, both markers indicate a more ancient intraspecies differentiation in *Z. japonica* than in *Z. marina*. Using the provisional molecular clock of ITS (0.8% to 2.0%; Bakker et al., 1995), we estimated that the split between Clade N and Clade S occurred 1.97–4.92 Ma (early Pleistocene to Pliocene). However, considering the limited number of variable sites used in the present study, whole genome data are needed for a more accurate time estimate.

Clear substructure was also detected within both Clade N and Clade S and genetically divergent subclades also displayed clear geographical patterns. Similar divergence between the northern and southern populations is also frequently reported in other marine species in the NWP (e.g. Harper et al., 2022; Krueger-Hadfield et al., 2017). These results taken together indicate that the present-day phylogeographic structure of *Z. japonica* may be closely related to historical and contemporary physical barriers that prevented gene flow among populations, resulting in vicariance and allopatric differentiation.

Glacial isolation of marginal seas is likely the primary driver for the deep genetic differentiation within *Z. japonica*. Many marine phylogeographic studies in the NWP have related significant intraspecific clades to geographically consistent glacial changes in marginal seas, implying that these basins served as separate refugia during glaciation (e.g. Hu et al., 2011; Liu et al., 2007; Xu & Chu, 2012). During Pleistocene glaciation episodes, the sea level in the East China Sea receded about 130–150 m below the present level at most, while the South China Sea was 100–120 m lower (Wang & Sun, 1994). This effectively reduced the East China Sea to an elongated trough (Okinawa Trough), while the South China Sea became a semi-enclosed inland sea connected to the Pacific through the Bashi Strait. The South China Sea was isolated from the East China Sea by the Dongshan land bridge that emerged between continental China and Taiwan Island during glaciation (Kimura, 2000; Zhao et al., 2017). During this time, the Sea of Japan/East Sea was almost totally isolated from the Pacific Ocean. Based on the geographic pattern of Clades N and S in *Z. japonica*, we suggest that Clade N originated from the Sea of Japan/East Sea and/or the Pacific Japan coasts, while Clade S originated from the South China Sea. Furthermore, the ancestors of the two clades must have experienced repeated contractions and expansions in later glacial–interglacial cycles and likely occupied a wide latitudinal range. Consequently, the divergence of subclades likely resulted from more recent isolation between their corresponding habitats.

Habitat discontinuity resulting from Yangtze River outflow has likely contributed to reinforcing or maintaining the historical separation, resulting in the allopatric distribution of *Z. japonica* populations along the coast of China. The abundant Yangtze River-derived mud deposit on the inner shelf, combined with contribution from other rivers, has created a large delta plain (3 × 10^4^ km^2^) and a muddy tidal flat along the coastline from the Northern Jiangsu Province (~35° N) to the Southern Zhejiang Province (30° N) over about 500 km wide (Zhu et al., 2008). A few studies have revealed the Yangtze River Estuary as a physical barrier for several intertidal molluscs (Dong et al., 2012; Li et al., 2021; Ni et al., 2015; Wang et al., 2015) or macroalga (Cheang et al., 2010) due to the unusual substrate and/or the effect of freshwater discharge. As *Z. japonica* could tolerate short-term exposure to zero salinity in situ (Kaldy & Shafer, 2013; Shafer et al., 2011) and survive in very muddy bottom (Zhang, Lin, et al., 2019), neither the salinity nor the substrate conditions seem to have limited the survival of *Z. japonica* in the Yangtze River Estuary. In contrast, permanent light deficiency probably is the primary limitation for the establishment of *Z. japonica* populations, which has also been referred to as the reason for the absence of other seagrass species in this area (Yang & Wu, 1981). Therefore, combined with the limited dispersal capability of *Z. japonica* and lack of stepping-stone populations, the broad coastline affected by the Yangtze River has acted as an unsurmountable geographic barrier hindering the postglacial dispersal and exchange.

Apart from that, natural selection may also have played an important role in the divergence between Clade N and Clade S. The distribution of the two clades was bounded by a prominent biogeographic boundary in the NWP, which extends from the Yangtze River Estuary in China to Jeju Island (Korea) and Southern Japan, separating the temperate and tropical/subtropical biotic regions (Lee et al., 2021; Liu, 2013; Ni et al., 2017). This pattern presumably reflected adaptation to colder or warmer environments by ancestral populations of the two clades since the early ice ages. Differences in life history between the temperate and subtropical *Z. japonica* populations also supported this hypothesis. For instance, temperate *Z. japonica* populations flower only once a year, from summer to autumn (Zhang et al., 2020), while the subtropical populations flower either twice a year (Nakaoka & Aioi, 2001) or once but earlier in spring (Qiu, 2021). Similar genetic divergence following long-term adaptation to different temperature regimes has been suggested in a number of intertidal benthonic animals in NWP, such as mitten crab (Xu et al., 2009), flathead mullet (Shen et al., 2011), sand lance (Han et al., 2012), gastropod (Zhao et al., 2017), mantis shrimp (Cheng & Sha, 2017), barnacles (Chang et al., 2017), pearl oyster (Takeuchi et al., 2020) and mussel (Lee et al., 2021). We suggest that early glacial isolation and long-term adaptation to distinct thermal conditions jointly drove the profound genetic differentiation between the N and S clades. It is important to note that the markers used in this and other genetic studies are neutral markers, which theoretically cannot reveal adaptive signals. Consequently, an extensive genomic analysis of nuclear genes is required to clarify whether these two clades have adapted in response to thermal differences or whether they represent two cryptic species in the NWP.

#### 4.1.2 | Secondary contact of Clades N and S

Sympatric distribution of the two *Z. japonica* clades was observed in four NWP populations (Yul, Nan, Hir and Tok). We interpret the sympatric occurrence as a result of secondary contact between formerly divergent clades, as postglacial range expansions might allow formerly isolated populations to have secondary contact at suture zones (Ni et al., 2014). Co-occurrence of ITS ribotypes of the two different clades within individuals from populations Yul, Nan and Tok suggests a recent hybridization between Clade N and Clade S (Liu et al., 2011). Our results suggest a northward expansion of Clade S from the South China Sea to the East China Sea facilitated by the Kuroshio Current, finally forming the present secondary contact zone. Northward range expansion is happening more frequently under global warming, as documented in marine snails, corals and fishes (Perry et al., 2005; Precht & Aronson, 2004; Zacherl et al., 2003). Similarly, the seagrass *Halophila nipponica*, which is primarily restricted to warm water areas of Japan, has been documented in temperate coastal regions of Korea recently, which is attributed to the increasing water temperature (Kim et al., 2009). Enhanced warming has been observed along the path of the Kuroshio Current in the last century due to a strengthening and poleward migration of the current (Wu et al., 2012). For *Z. japonica*, we propose that the species range and the secondary contact zone of the two clades may continue this northward shift in response to climate warming.

In addition, an interesting distribution pattern of Clades N and S was observed along the western coast of Japan within the Sea of Japan/East Sea, where the relatively southern population Oha belongs to Clade N while the relatively northern populations Lida and Nan are dominated by Clade S. One possible explanation is that Clade S was transported to Lida and Nan via bird-or human-mediated activities. Another reason for the dominance of Clade N in the population Oha could be its special geographic features. The population Oha was relatively isolated from the open ocean, which might make it difficult for the spread of Clade S. More comprehensive sampling will be helpful to further clarify the distribution and dispersal mechanism of the two clades within this area.

### 4.2 | Invasion history in the nonnative range

#### 4.2.1 | Introduction through oyster imports

Inadvertent and deliberate species introductions have long been facilitated by anthropogenic activities such as shipping, aquaculture and aquarium hobbyists (Wonham & Carlton, 2005). It has been long hypothesized that vegetative or seed-bearing shoots of *Z. japonica* were introduced to America as packing materials for transportation of the Pacific oyster, *Crassostrea gigas* (Bigley & Barreca, 1982; Harrison, 1976; Harrison & Bigley, 1982; Sayce, 1976). Beginning in the early 1900s, Pacific oysters were imported from Japan for aquaculture purposes, which peaked around the 1950s (Quayle, 1988; Scagel, 1956). Several other nonnative species were confirmed to have been introduced to PNA by oyster imports, including two algal species (Krueger-Hadfield et al., 2017; Scagel, 1956), the Japanese oyster drill (Lindsay & Simmons, 1997) and the Asian mud snail (Miura et al., 2006).

**FIGURE 6:**
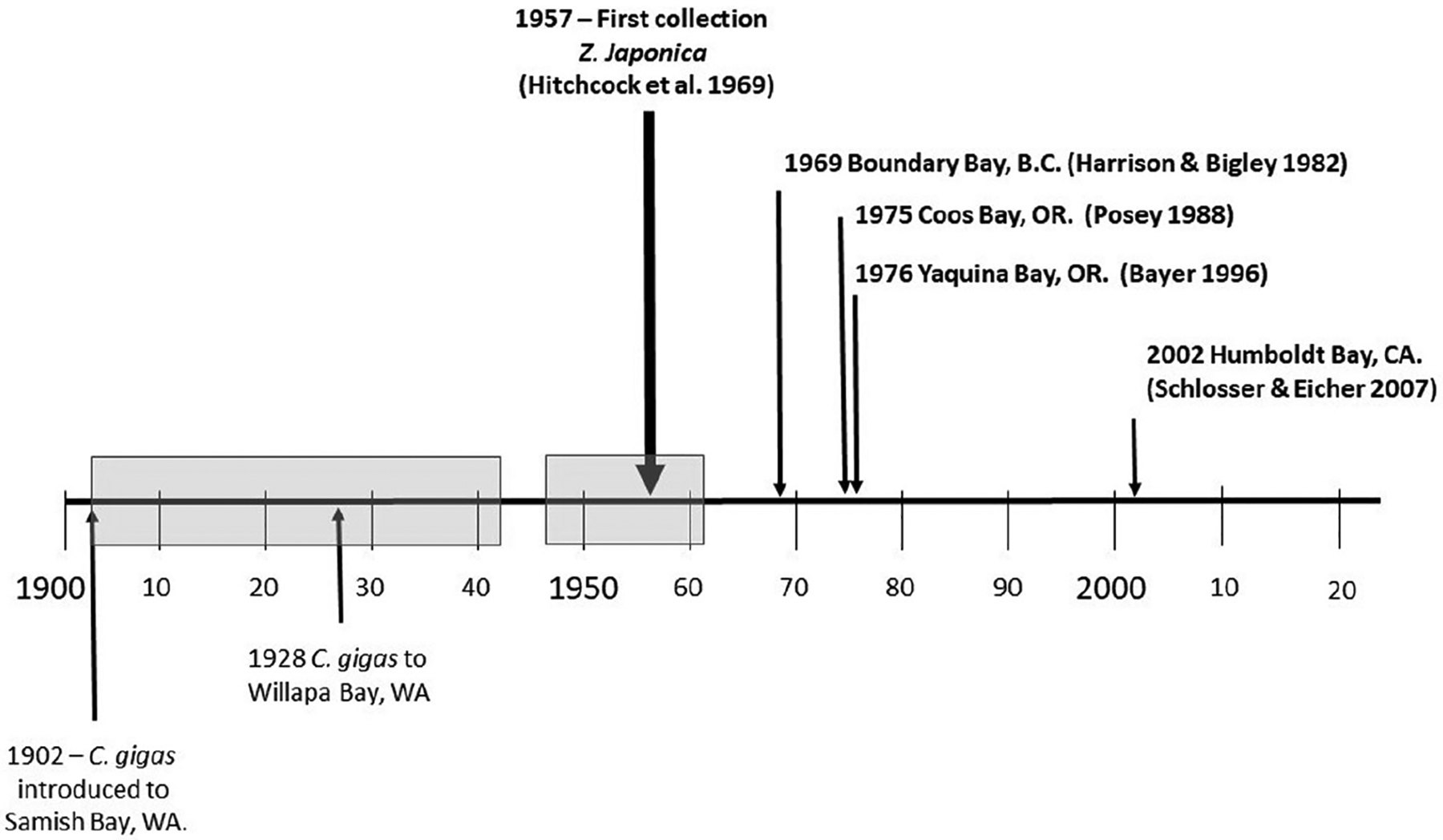
Timeline of initial introduction of *Crassotrea gigas* to Samish and Willapa Bays in Washington and documented first records of *Zostera japonica* collection across North American locations. The grey boxes represent the period of oyster and oyster spat imports from Japan to Washington State. From 1942 to 1946, there were no imports due to WWII.

We plotted an approximate timeline for the first introduction of Pacific oyster compared to the first reports of *Z. japonica* in various sites in PNA (Figure 6). This timeline illustrates some important coincidences in terms of both time and locations between the occurrence of *Z. japonica* and oyster that appear to be corroborated by the genetics results. The first collection of *Z. japonica* was in Willapa Bay, USA, in 1957 (den Hartog, 1970). In addition, the two bays with the largest populations of *Z. japonica* were northern Puget Sound and Willapa Bay (Harrison & Bigley, 1982). Coincidently, the first sites of *C. gigas* cultivation in Washington State were Samish Bay (northern Puget Sound adjacent to Padilla Bay) and Willapa Bay. In 1902, the first 500 cases of oysters were shipped from Hiroshima, Japan, to Samish Bay in Bellingham, WA, USA. In 1928, the first introduction occurred when 40 cases of oyster seed were test planted in Willapa Bay, WA (Sayce, 1976). Oysters were imported from a variety of places in Japan including Kobe, Tokyo, Kanazawa, Watanoha and Akkeshi (Hori, 1947) and Sendai Bay, Miyagi prefecture (Sayce, 1976). The timing of when oyster seed importation ended is vague. Lindsay and Simmons (1997) indicated imports ceased in 1979, while Lavoie (2009) suggested imports had practically ceased by 1961.

#### 4.2.2 | Possible origins and secondary dispersal

All analyses supported that the population Kat from the Miyagi prefecture located on the central Pacific coast of Japan is the most likely source for the nonnative populations. However, significant genetic structure and absence of IBD within the nonnative range suggested multiple source populations. *Zostera japonica* populations in Padilla and Willapa, which are close to the original oyster introduction sites, represent two unique genetic clades. Thus, it is likely that there might have been at least two independent introduction events from different sites located in Pacific Central Japan. Significantly lower genetic diversity in the nonnative than the native range indicates a bottleneck or founder effect in the early stage of introduction.

The genetic affinity within the two sub-clades of the USA suggested that Samish Bay and Willapa Bay may have been bridgeheads for secondary dispersal of *Z. japonica* to adjacent areas. The noticeable migration rate between Willapa and Yaquina Bay might indicate a very recent or ongoing dispersal from the former to the latter. A variety of vectors have been hypothesized to account for the expansion of *Z. japonica*, including transport of reproductive material by currents and boats, transfer of viable fragments and transport of viable seed by waterfowl (Shafer et al., 2014). Several waterfowl species in North America are known to feed heavily on *Z. japonica* seeds, leaves and rhizomes (Baldwin, 1994; Baldwin & Lovvorn, 1994), and thus it is likely that waterfowl could be a major vector in the establishment of new *Z. japonica* populations (Shafer et al., 2014), which is also reported for several aquatic plants (Darwin, 1859; Ganter, 2000; Ridley, 1930; Rivers & Short, 2007) and the congener *Z. noltii* (Coyer et al., 2004). The lack of significant IBD, which is different from that in the naturally colonized *Z. marina* populations in the Pacific (Duffy et al., 2022), also supports our hypothesis of bird-mediated dispersal.

We would expect that *Z. japonica* in Humboldt Bay could spread further southwards, and our predicted southern boundary could be Baja California, which has plenty of habitats that are suitable for *Zostera*. It is clear from our results that *Z. japonica* populations in PNA are derived from Clade N, and the temperature in Baja California is similar to that in the native southern boundary of Clade N. However, it cannot be ruled out that *Z. japonica* could spread further southwards, as the southern populations might be better adapted to warmer temperatures and higher irradiances than the northern ones (Shafer et al., 2008). Similarly, DuBois et al. (2012) have also found local adaptation to temperature in American *Z. marina* populations within a fine scale (2–12 km). Thus, clarifying local adaptation and species resilience of *Z. japonica* to global change would be helpful in predicting its potential spreading limit.

### 4.3 | Implications for the conservation of *Z. japonica*

The two deeply divergent clades of *Z. japonica* corresponding to different climate zones (temperate vs. tropical–subtropical) and distinct reproduction strategies (flowering frequency, seedling recruitment frequency and phenology) in its native range represent two evolutionary significant units (ESUs) (Ryder, 1986) that should be conserved separately. We argue that conservation policies should target the maintenance of *Z. japonica* across its entire range in order to maintain as much of the important genetic building blocks as possible since populations exhibit high genetic differentiation and weak (or absent) genetic connectivity between most population pairs. The relatively high genetic differentiation within the native range probably resulted from the limited dispersal potential of this species (Jiang et al., 2018) combined with increased habitat discontinuity. In comparison, the southern populations, with lower genetic diversity, lower *N*_e_ and higher *F*_ST_, were likely to be more vulnerable to disturbances and local extinction. Thus, conservation efforts are particularly urgently needed for the southern populations, especially those with small effective population sizes (<50). The populations with high genetic diversity should also be prioritized for conservation as they can be ideal donors for restoration. Restoration should be undertaken carefully, taking genetic diversity and the spatial population structure into account, which is related to adaptation to local environments (Kurokochi et al., 2015). On a positive note, despite the ongoing degradation of many *Z. japonica* populations, mainly due to habitat destruction and fragmentation, the genetic potential for recovery may be locally available. Thus, conservation and management approaches are needed to improve water quality, habitat continuity and connectivity between populations.

## 5 | CONCLUSIONS

This study provides a full view of both the phylogeographic and invasion history of the seagrass *Z. japonica* throughout its native and nonnative range. In the native range, deep differentiation and clear secondary contact between the northern and southern populations were detected, possibly driven by historical and present physical isolation and temperature gradients. In the nonnative region, at least two independent invasion events were found from the central Japanese Pacific coasts, with the invasive populations experiencing leapfrogging dispersal and potential for further expansion in PNA. These results not only help to understand the underlying evolutionary potential of *Z. japonica* following drastic environmental changes (e.g. climate change) but also provide valuable insights for conservation and restoration.

## Supplementary Material

Supplement1

Supplement2

## Data Availability

Microsatellite data that support the findings of this study can be found in the Figshare data repository at https://figshare.com/s/ef930a0dfa16f2fad94d. DNA sequences can be found in GenBank (Accession nos OQ 826013–OQ826067 and OQ835572–OQ835574).
