# Supplementary material for: Population genetic patterns across the native and invasive range of a widely distributed seagrass: Phylogeographic structure, invasive history and conservation implications
Source: Divers Distrib. Author manuscript; Available in PMC 2025 Mar 1. (PMC10953713; doi:10.1111/ddi.13803)
Supplement: Supplement2 [file NIHMS1976875-supplement-Supplement2.doc]

**Appendix 2**


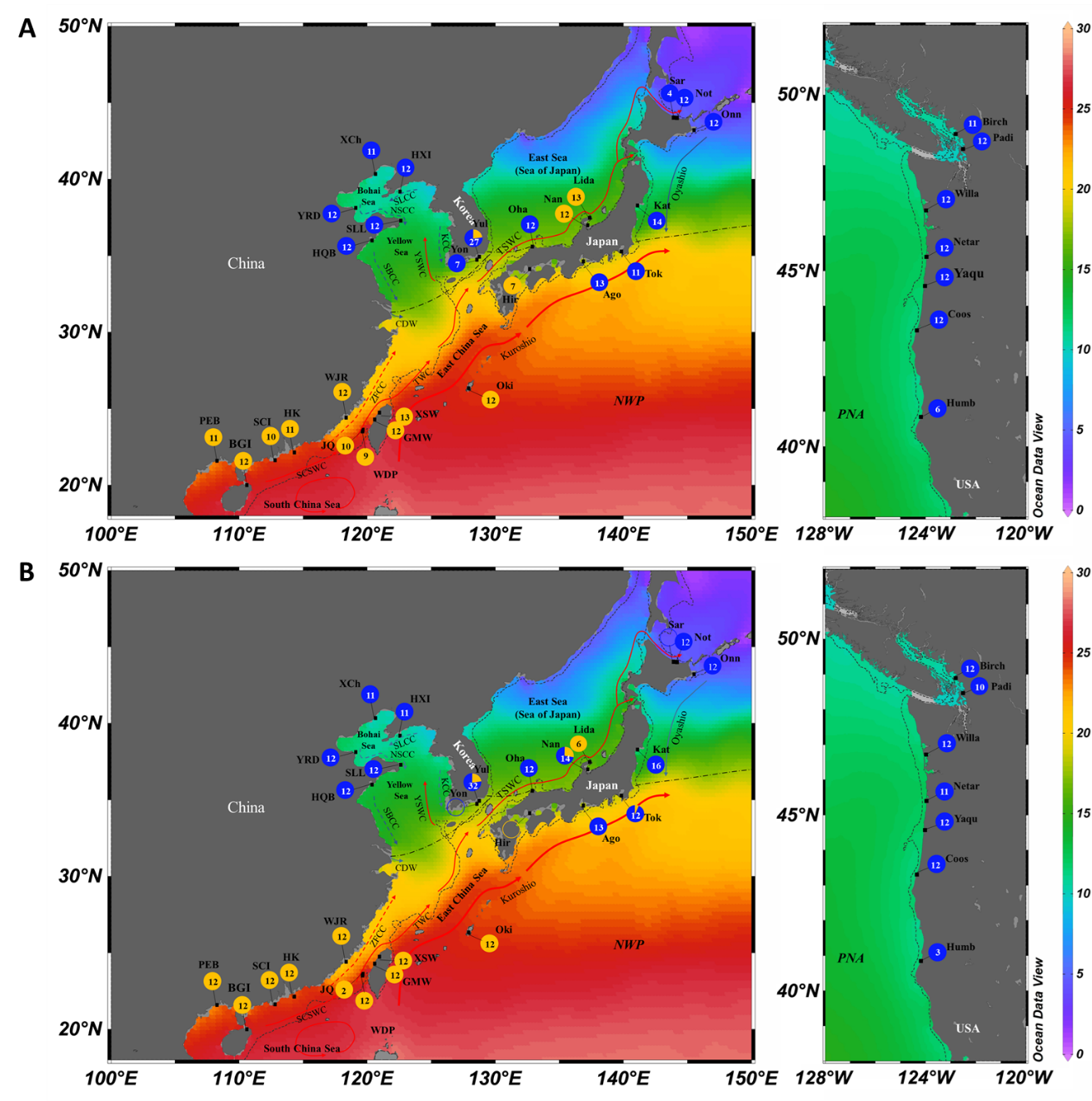


**Figure S2.1** The distribution of the two clades of *Zostera japonica* inferred from *matK* (A) and ITS (B). The sample sizes were represented as the numbers in the circles. The southern and northern clades are represented by orange and blue colored circles, respectively. The map also presents the annual mean sea surface temperature distribution across the study area, as derived from the World Ocean Atlas 2018 dataset (Locarnini et al., 2019) using Ocean Data View software (Schlitzer, 2015). Currents in summer are depicted with arrows and include the South China Sea Warm Current (SCSWC), Taiwan Warm Current (TWC), MinZhe Coastal Current (ZFCC), Changjiang Diluted Water (CDW), Subei Coastal Current (SBCC), Lubei Coastal Current (LBCC), Liaonan Coastal Current (LNCC), Yellow Sea Warm Current (YSWC), and Tsushima Warm Current (TSWC). The shelf outcrop of the last glacial maximum is represented by a dotted line. The dash/dotted line separates two biogeographic regions: the North Pacific Temperate Biotic Region (above the line) and Indo-West Pacific Warm Water Biotic Region (below the line).


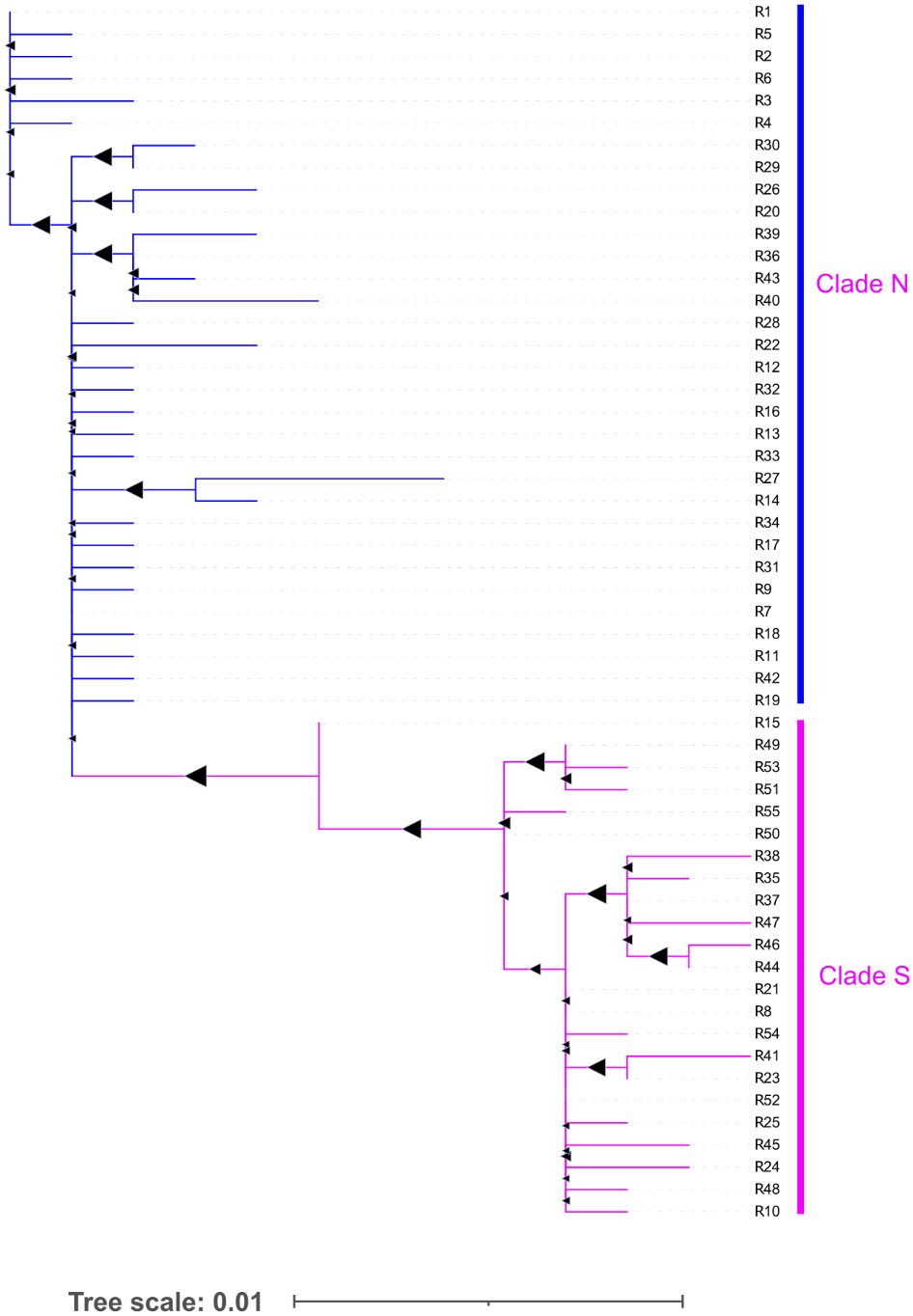


**Figure S2.2** Maximum likelihood (ML) tree based on ITS sequences for *Zostera japonica* populations across its native and non-native range.


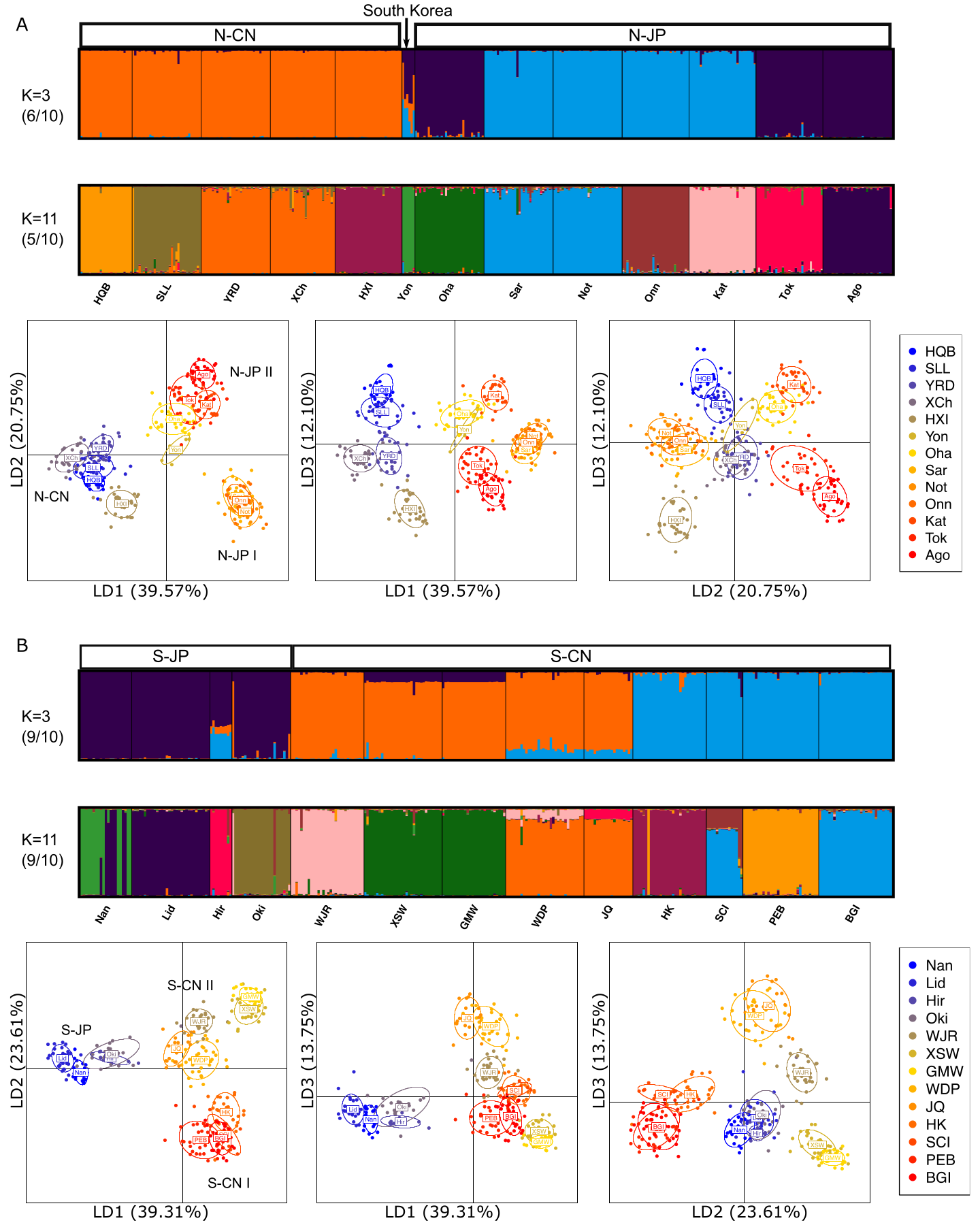


**Figure S2.3** Substructure of the native *Zostera japonica* populations within Clade N (A) and S (B).


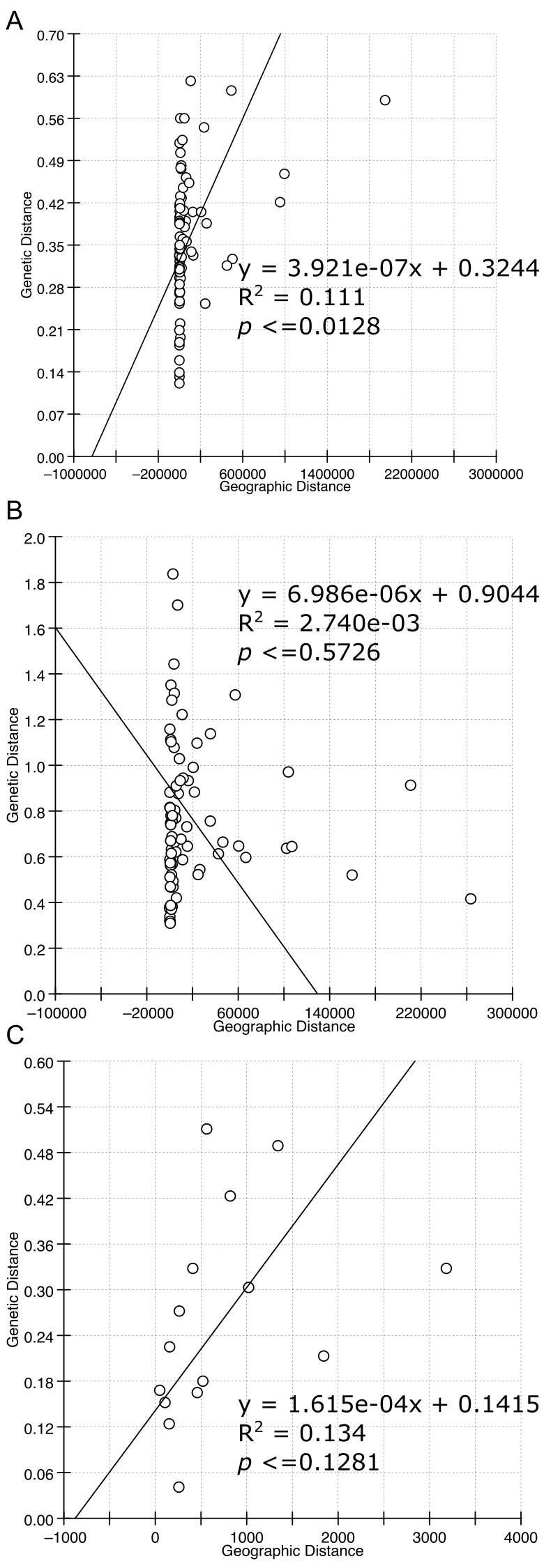


**Figure S2.4** Isolation by distance for *Zostera japonica* populations in Clade N (A), Clade S (B), and USA (C)


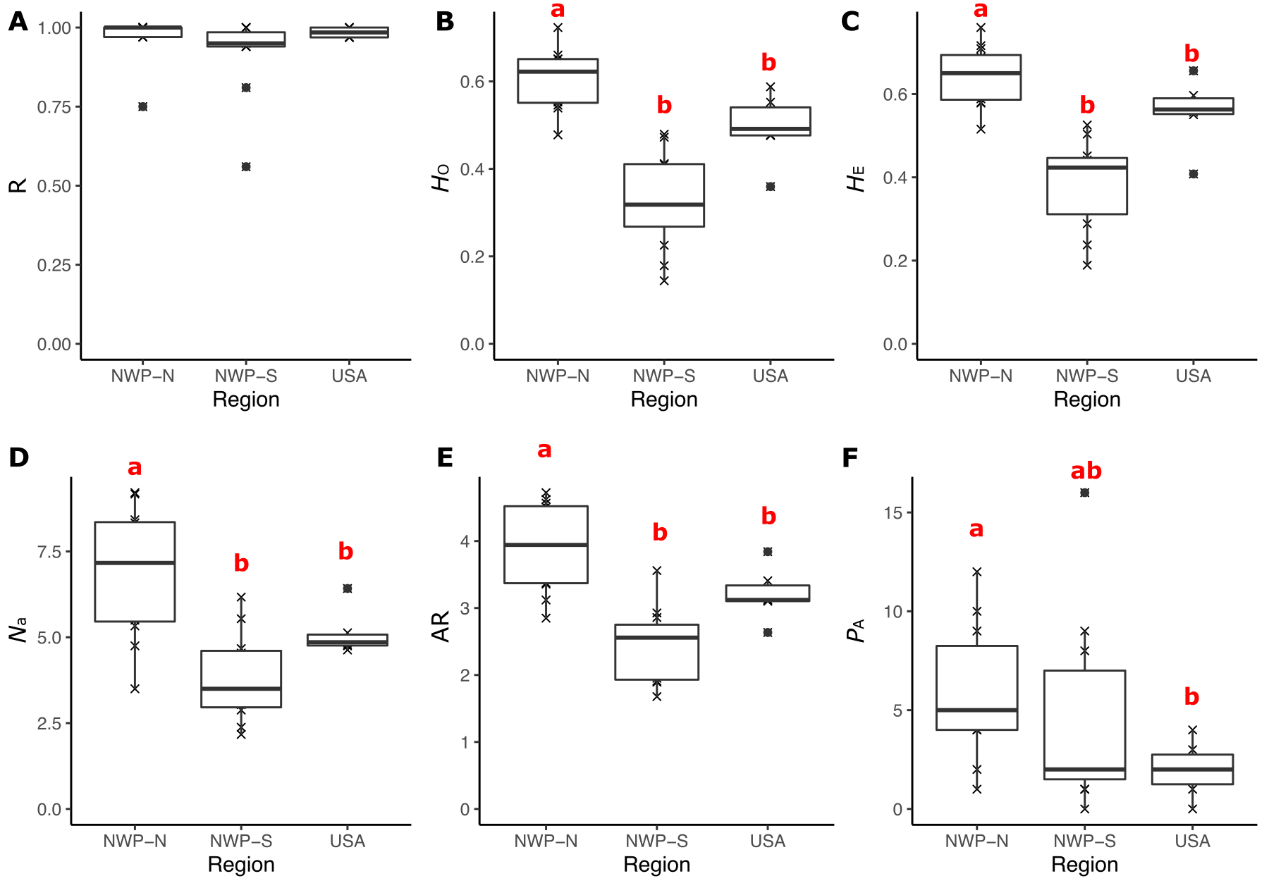


**Figure S2.5** Genetic variation indicators (A-F) in three groups (NWP-N, NWP-S, USA) of *Zostera japonica* populations. A, genotypic richness (R); B, observed heterozygosity (*H*_O_); C, expected heterozygosity (*H*_E_); D, private alleles (*P*_A_); E, the number of alleles per locus (*N*a); F, allelic richness (*AR*). NWP-N, the native populations in Clade N; NWP-S, the native populations in Clade S; USA, the non-native populations.

**Data Availability Statement**

Microsatellite data that support the findings of this study can be found in the Figshare data repository at https://figshare.com/s/ef930a0dfa16f2fad94d. DNA sequences can be found in GenBank (Accession nos OQ 826013-OQ826067, OQ835572-835574).
